# Supplementary material for: 3′quant mRNA-Seq of Porcine Liver Reveals Alterations in UPR, Acute Phase Response, and Cholesterol and Bile Acid Metabolism in Response to Different Dietary Fats
Source: Genes (Basel). 2020 Sep 18;11(9):1087. doi: 10.3390/genes11091087 (PMC7565913; doi:10.3390/genes11091087)

Rapeseed oil vs Beef tallow, the more intense red colour, the lower p-value.

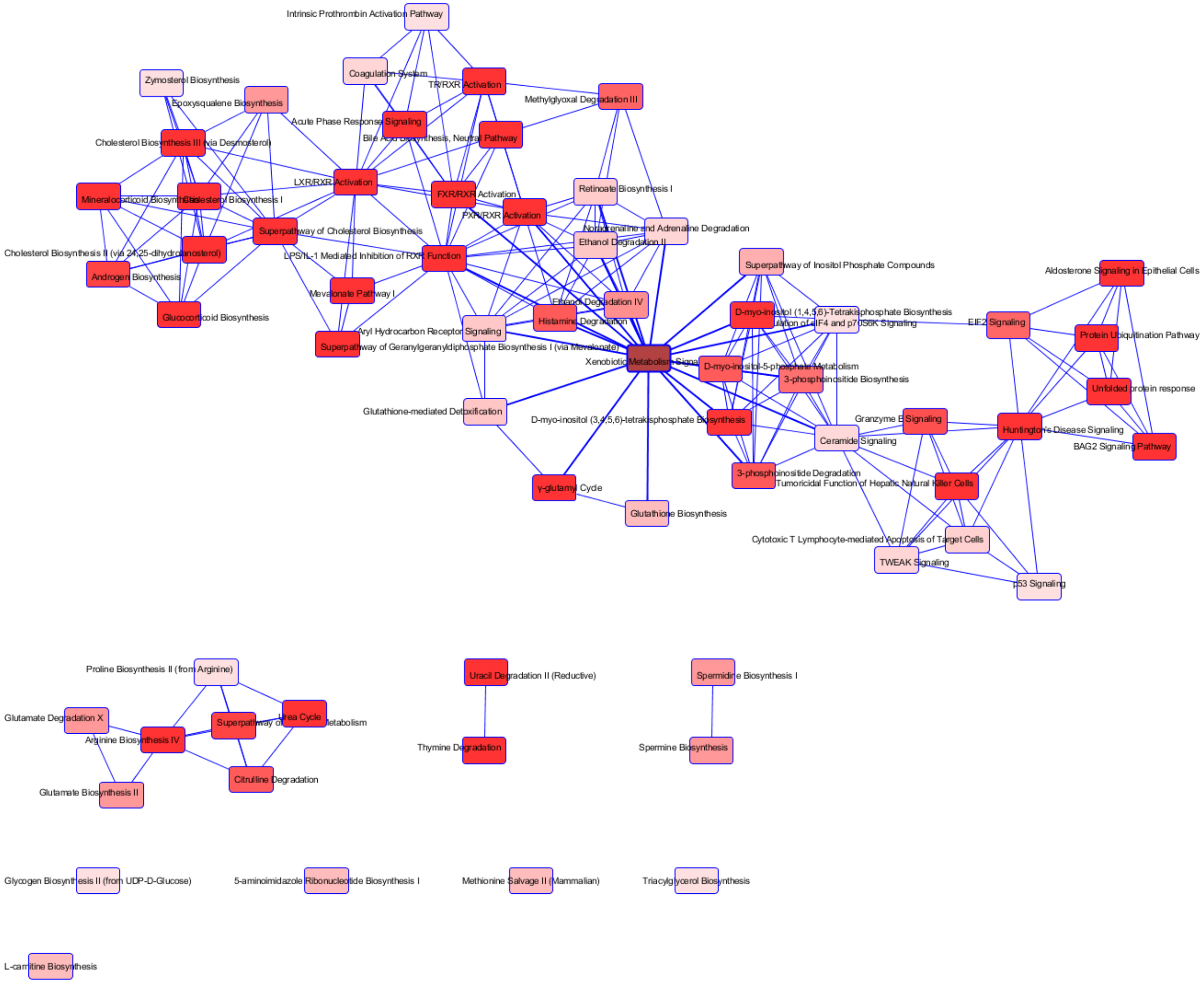

Rapeseed oil vs Coconut oil, the more intense red colour, the lower p-value.

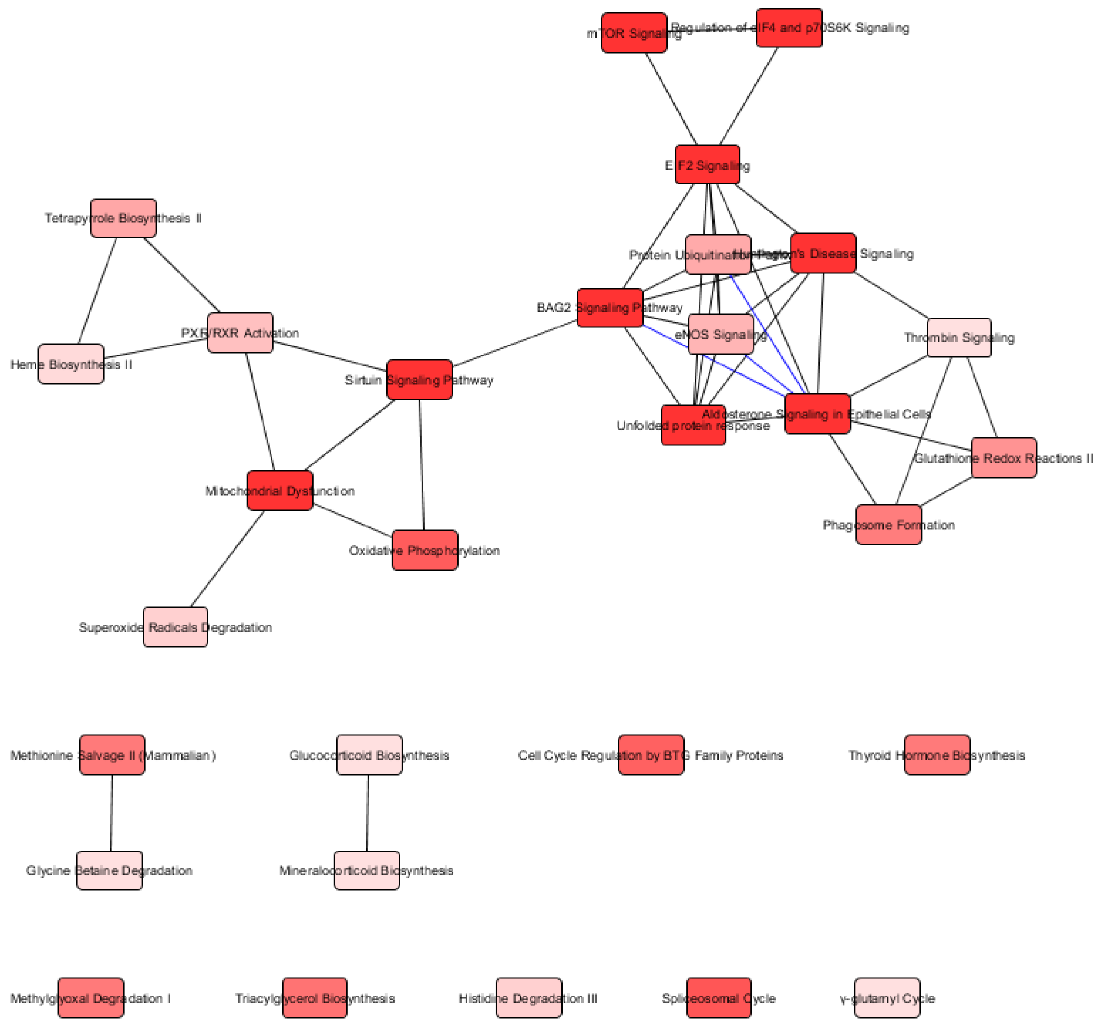

Beef tallow vs Coconut oil, the more intense red colour, the lower p-value.

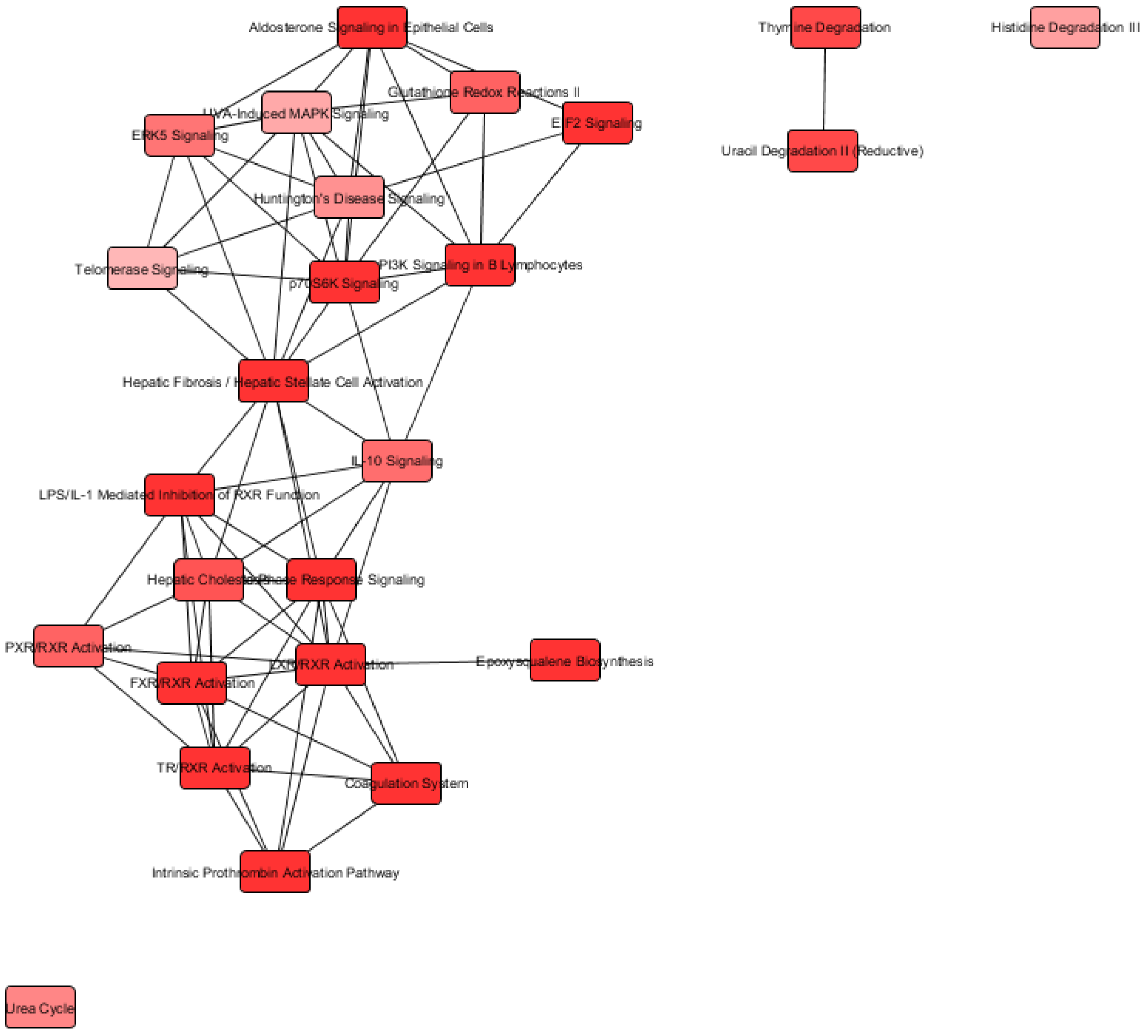

Supplement: Supplementary file 1 [file genes-11-01087-s001.zip › Supplementary figure 2..pdf]
