# Supplementary material for: 3′quant mRNA-Seq of Porcine Liver Reveals Alterations in UPR, Acute Phase Response, and Cholesterol and Bile Acid Metabolism in Response to Different Dietary Fats
Source: Genes (Basel). 2020 Sep 18;11(9):1087. doi: 10.3390/genes11091087 (PMC7565913; doi:10.3390/genes11091087)
Supplement: Supplementary file 1 [file genes-11-01087-s001.zip › Supplementary figure1.pdf]

Rapeseed oil vs Beef tallow
MA plot

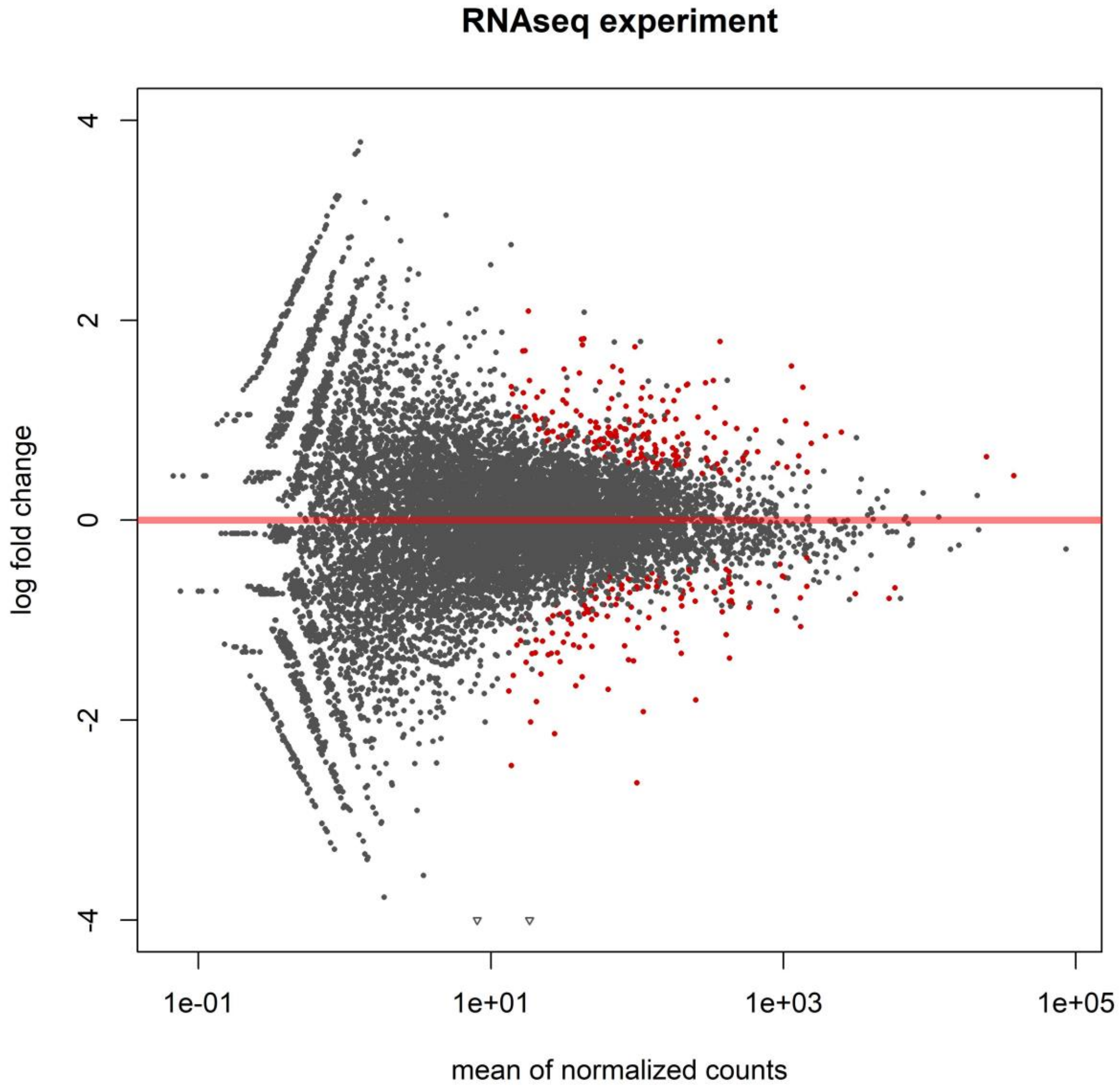

Rapeseed oil vs Beef tallow

PCA plot

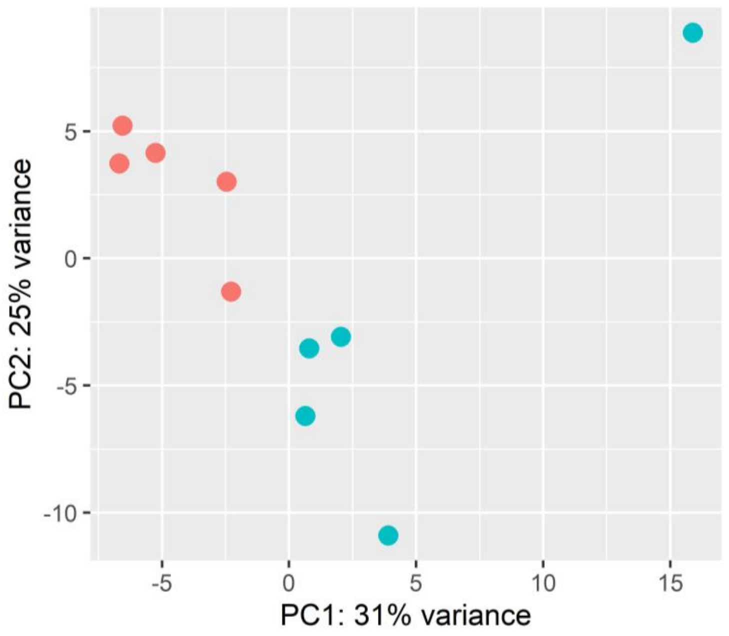

Rapeseed oil

Beef tallow

Rapeseed oil vs Coconut oil

MA plot

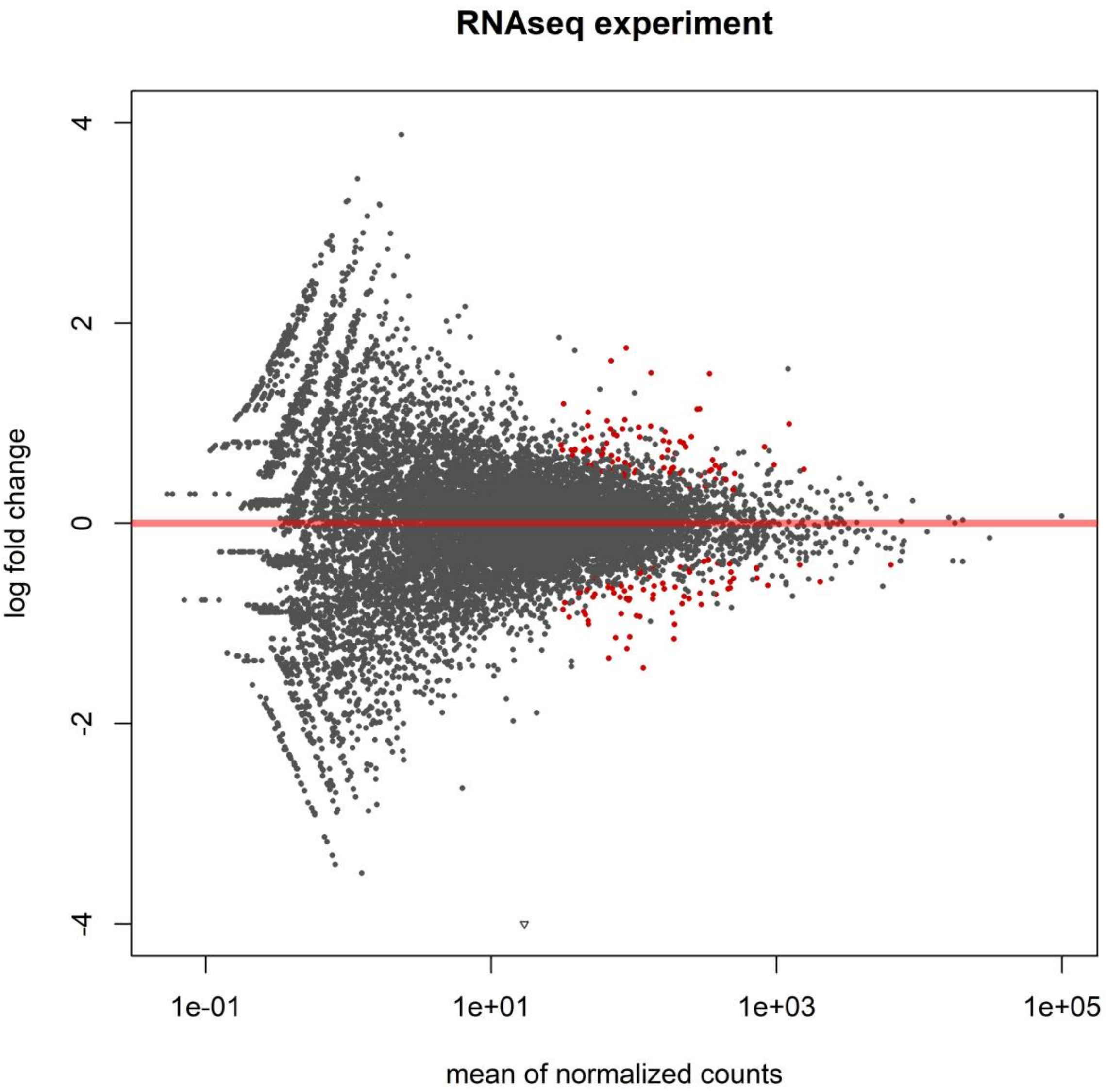

Rapeseed oil vs Coconut oil

PCA plot

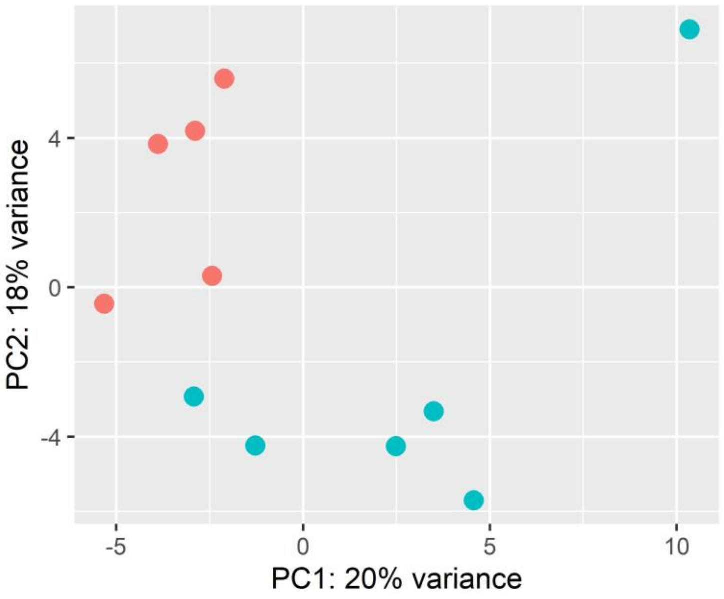

Rapeseed oil

Coconut oil

Beef tallow vs Coconut oil

MA plot

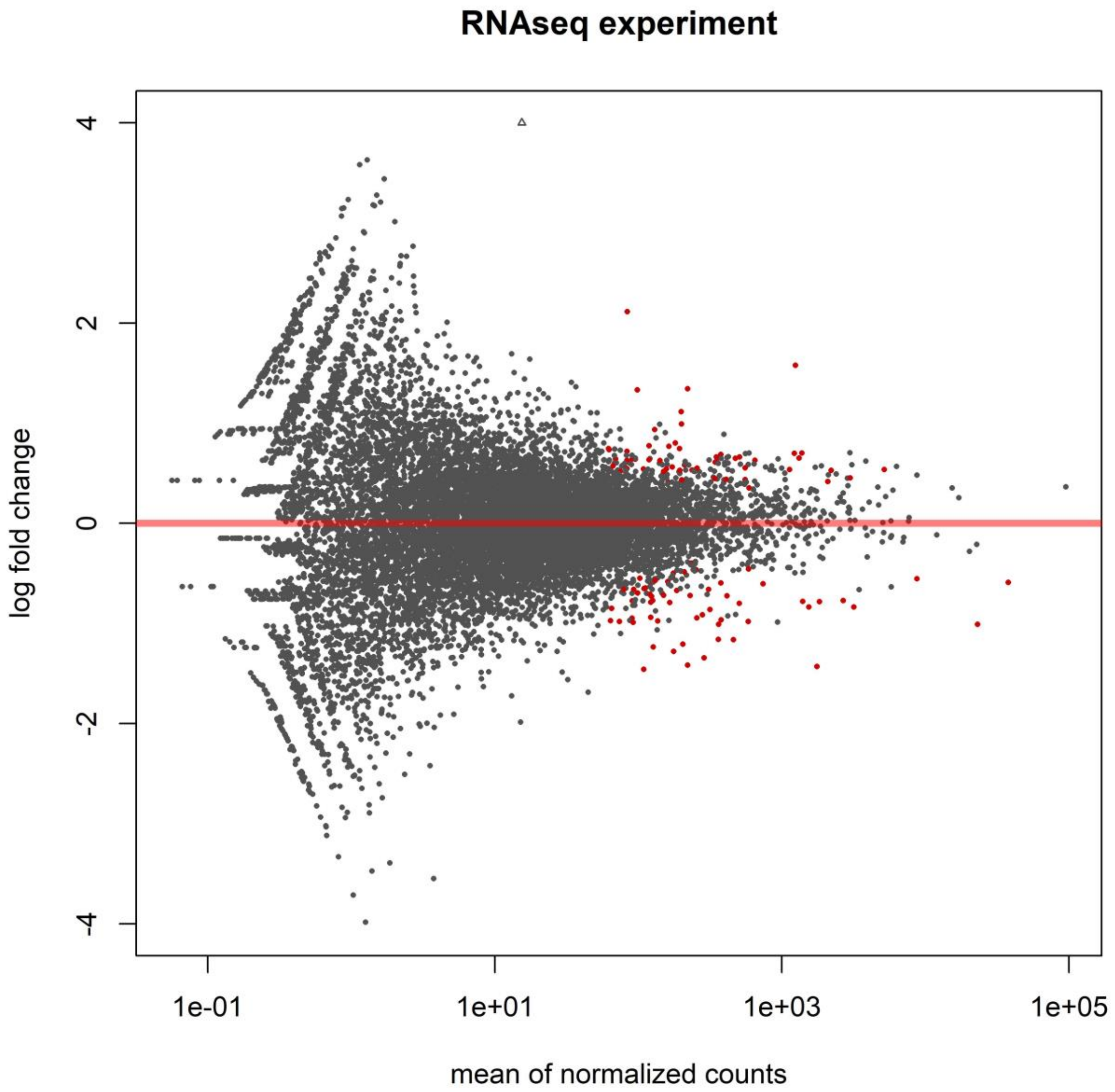

Beef tallow vs Coconut oil

PCA plot

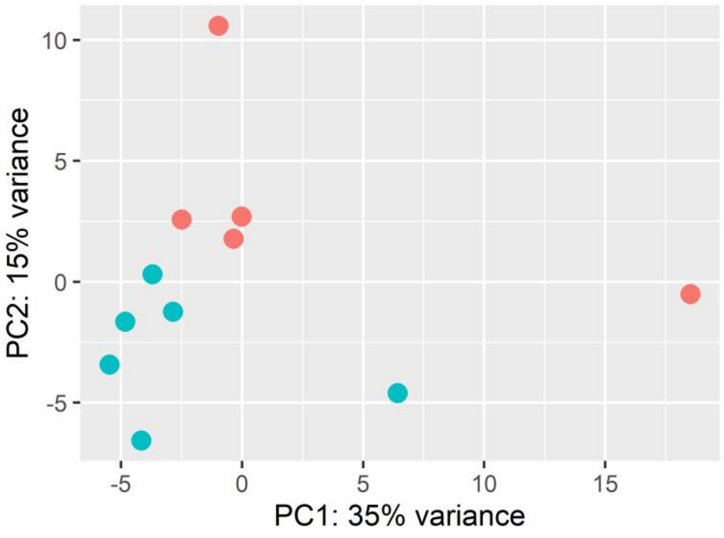

Beef tallow

Coconut oil
